# Progress in Medicine

**Published:** 1894-10-06

**Authors:** 


### Progress in Medicine.

### ANÆSTHETICS.

A great amount of labour has recently been undertaken by various investigators with a view to clearing up some of the more obscure and contentious points in connection with anæsthetics, and much valuable clinical experience, sometimes dearly bought, renders it desirable to collect and review the more important facts brought to light.

**Chloroform.**—In a letter on the Hyderabad Chloroform Inquiry[1] we find that the Commission found that—" (1) Chloroform and shock are incompatibles. It was found to be impossible to stop the action of the heart by any form of shock under chloroform. (2) In death by chloroform the respiration always stops before the heart, which beats effectively for a considerable time after the breathing has ceased. (3) The lowering of the blood pressure which is produced by the narcotic action of chloroform is harmless, and cannot be due to weakening of the heart. (4) Chloroform anæsthesia is free from risk. As long as the breathing is natural and regular, and the inhalation is not continued after anæsthesia is complete, over-dosing with chloroform is impossible. (5) The pulse is never appreciably affected by chloroform, except through interference with the breathing or over-dosing."

The profession, not being satisfied with the experiments of the Hyderabad Commission as to the absence of direct heart failure, further experiments were undertaken by Hare and Thornton and by Gaskell and Shore. The former substantiated those of the Commission, but the latter, by cross-circulation experiments, expressed their opinion that the fall of blood pressure which usually accompanies chloroform anæsthesia is mainly due to a direct weakening action of chloroform on the heart. A second series of cross-circulation experiments was completed at Hyderabad by Lawrie, with the result that when chloroform was sent to the heart alone it produced no effect whatever, but that when it was sent to the brain alone it produced its usual sequel of events, viz., lowering of the blood pressure, with first anæsthesia, then stoppage of the respiration, and then death.

The cross-circulation experiments thus established the following additional facts: " (1) Chloroform has no direct action on the heart or circulation. The heart can only be affected under chloroform by interference with the breathing or by overdosing, which is practically the same thing. Accordingly it must be futile and dangerous to take the pulse as a guide to its effect. (2) The lowering of the blood pressure, which is caused by an effective dose of chloroform, is due entirely to its direct narcotic action on the brain, and is a safeguard to the heart by dilating the smaller arteries and facilitating the flow of blood from the arterial to the venous system, thus diminishing its work and relieving it of strain."

These investigations support Symes' principles of chloroform administration. Although the results of the cross-circulation experiments of Gaskell and Shore are diametrically opposed to those of Lawrie, they still recognise the great importance of watching the respiration during the administration of chloroform.

In a demonstration at the London Hospital Lawrie[2] says, " Our creed about chloroform is a very simple one. The anæsthetic is administered by inhalation and during the inhalation there are two things to avoid, (1) the inhalation or in-take of an overdose, and (2) interference with the breathing. In order to avoid these evils, we must not be guided by the circulation but entirely by the respiration."

At a meeting of the Royal Medical and Chirurgical Society Lawrie[3], in investigating the mode of death in uncomplicated chloroform poisoning, refers to the important bearing in this connection of Mr. Horsley's experiments on bullet wounds of the head. The effect of a bullet traversing the cranial cavity at moderate velocity is, at first, complete arrest of the respiration and a slight fall of central blood pressure, followed a little later by a remarkable rise in blood pressure until the normal tension is exceeded. These observations prove that the first cause of death is not arrest of the heart and syncope as the heart goes on beating, although respiration has completely stopped. Furthermore, artificial respiration will bring about recovery These observations are considered seriatim, "(1) In bullet wounds respiration is arrested suddenly, under chloroform gradually, although under certain circumstances it may be arrested so rapidly as to amount practically to a sudden stoppage. (2) In death from chloroform and bullet wound, stoppage of the respiration is followed by gradual failure of the heart. (3) In chloroform poisoning the arrested circulation may frequently be restored by artificial respiration, and if death ensues post-mortem appearances are those of paralysis of the respiratory centre and not of asphyxia. This is precisely what Mr. Horsley states with regard to death from bullet wounds of the brain. (4) In death from chloroform the respiratory centre is narcotised or paralysed from the commencement, and is never stimulated at first as it is in asphyxia. This remark applies equally to bullet wounds, except that the narcosis of the respiratory centre is gradual in chloroform poisoning and sudden in bullet wounds of the brain. (5) The proper thing to do in chloroform poisoning is to employ artificial respiration rather than the giving of stimulants. These again are Mr. Horsley's own words with regard to bullet wounds of the brain. (6) In chloroform poisoning the narcosis may be complicated by vagus stimulation, which produces marked slowing of the heart. The same thing occurs in bullet wounds of the brain. In the former we regard this slowing of the heart as a safeguard in that it tends to diminish the blood supply which is conveying the poison to the brain and producing the fatal narcosis. In the latter the slowing of the heart may also be a safeguard in that it tends to arrest the hæmorrhage which is producing the fatal compression. We now know that bullet wound of the brain no less than chloroform poisoning causes death by respiratory arrest, and that it is not due either to syncope or asphyxia. No doubt in death from chloroform the process may be complicated by asphyxia, but it is important to bear in mind that the only danger of asphyxia under chloroform is to cause an increased in-take of the anæsthetic and very rapid narcosis of the respiratory centre, a narcosis which may be so rapid and permanent as to appear very like sudden death." At this meeting Shaw and Gaskell severely criticised Lawrie's cross-circulation experiments, referring to the fact that he had only produced three tracings, and that these were not sufficient to prove his point with reference to the action of chloroform on the heart, which differed from the results obtained by heir experiments.

**Salicylide Chloroform.**—All chloroformists are agreed that the purity of the drug is of paramount importance, and from time to time new preparations appear. Fowler[4] describes Professor Anschütz's salicylide chloroform, in which salicylic anhydride is used for purposes of purification, whereby it is said an absolutely pure chloroform results. The points to note in connection with this new preparation are (1) Its rather pleasant aromatic odour in place of the pungent odour of ordinary chloroform, and consequently less hesitancy on the part of the patient in respiring it. (2) The stage of excitement and struggling is much less marked than usual. (3) The length of time required to render a patient completely anæsthetic is longer than with the ordinary drug, taking about ten minutes usually. (4) Patients recover consciousness more quickly, and there is a marked absence of the usual after effects of chloroform. Dr. Fowler recommends that the chloroform should be given drop by drop, keeping up the anæsthesia by a small dose frequently repeated.

**Chloroform in Organic Heart Disease.**—The Hyderabad Commission have shown us that it is quite safe to give chloroform in heart disease, and Clarke[5] and Giffen[6] are of the same opinion, and maintain that it is far safer to give it than not, the shock of the operation and the struggling from pain both of them more seriously affecting the heart than the anæsthetic. Chloroform is the best anæsthetic in most of these cases, but if there be any suspicion of fatty or other degeneration of the heart muscle, Dr. Giffen says we should be forced to employ ether.

**Deaths under Anæsthetics and Means of Averting it.**—Out of twelve deaths reported in various journals during the past few months the anæsthetic in nine cases was chloroform, in one the A.C.E. mixture, in one a mixture of chloroform and ether, and in one first chloroform and then ether. Of these cases half died before the operation was commenced, in the others the operation was advanced to various stages, but in hardly any of the cases was it completed. In two of these it is definitely stated that the pulse ceased before the respiration, and in four others death is attributed to syncope, one of these having a fatty heart, as discovered at the post-mortem (this was the case in which ether was administered after a little chloroform had been given). Kirk[7] also describes a case of probable syncope from chloroform, the patient recovering. In the remainder of the cases the respiration is said to have ceased first, and in some the pulse is described as beating for some time after, and in others as not being felt after respiration had ceased. With the exception of the case described above with fatty heart nothing was found post-mortem to account for death in any of the cases. The usual attempts at resuscitation were practised in all, artificial respiration being the first and most important, but in only two cases after the respiration had ceased was there any voluntary effort at respiration, and this only repeated a few times, and then ceased again. It is pointed out[8] in connection with one of these cases that in emphysema of the lungs it is very easy to give an overdose of chloroform, as expiration being slight and inefficient, little or none is eliminated from the pulmonary mucous membrane.

**Strychnine.**—A remedy that has been lately recommended in the treatment of chloroform narcosis is the administration of strychnine hypodermically. Washburn[9] describes a case of chloroform swallowed with suicidal intent successfully treated in this way; and Blanks[10] says that, when used for this purpose, it is no use giving small doses—" not less than a twentieth of a grain, and in desperate cases one-tenth, or even one-fifth, of a grain may be given with impunity." With regard to electricity in chloroform narcosis, Blanks[11] is of opinion that a strong current should not be applied to the pneumogastric nerve, owing to its cardio-inhibitory fibres; whereas Rockwell[12] says his experience is that ordinary external applications of the faradic current to the vagus "affect the inhibitory fibres that go to the heart in no such degree as they affect the accelerating fibres, which in great measure constitute the branches that supply the respiratory organs." He further adds,[13] "electricity does good in increasing respiratory activity, the strength of current for this purpose being insufficient to materially interfere with the movements of the heart through its action on the inhibitory fibres of the pneumogastric that control it." As an additional precaution in the administration of chloroform, Drs. Langlois and Mamange[14] recommend the injection of sparteine and morphine in doses of $\frac{2}{20}$ to $\frac{1}{5}$ grain of the former and $\frac{1}{6}$ grain of the latter, fifteen minutes before anæsthetisation, in order to prevent the initial cardiac syncope which sometimes supervenes. They have practised this procedure in 120 cases, and found the pulsations of the heart were invariably and uninterruptedly regular and full. Drs. Dastre and Morat had previously advised a mixture of atropine and morphine for the same purpose. It is assumed that these drugs restrict the inhibitory action of the pneumogastrics on the heart.

In combatting chloroform collapse, Sir B. W. Richardson says that the "artificial respiration, as performed by Sylvester's and Marshall Hall's methods, or by any other that causes active movement of the body, must be injurious, as tending to reduce the power of the feebly-moving heart." He recommends mouth-to-mouth insufflation, or the use of his double-acting hand bellows, which exhaust by one bulb and fill by another; he also strongly advises the use of air slightly charged with ammonia vapour. As an addition to artificial respiration, he advocates inversion of the body. He also makes a suggestion that when the heart still beats, but has not power to force blood from one side to the other, the transfusion of blood kept fluid by ammonia might be useful. He concludes by saying "that in death from chloroform gentle artificial respiration by simple inflation of the lungs through the nostrils, with the body inverted, is the only rational means of resuscitation."

Warholm[15] accidentally discovered the value of vinegar in relieving the after effects of chloroform. He has used it in thirty cases, and not only were the nausea and vomiting relieved, but also the distressing headache. On the patient being brought back to bed, a compress saturated with vinegar is placed over his nose, and left there till he comes round, or longer if necessary.

**Methods of Administration.**—Notwithstanding the Hyderabad Commission there still remain experimenters and anæsthetists who consider that chloroform has a direct action on the heart, and that therefore the pulse should be watched in addition to the breathing during anæsthetisation, and certainly cases have been reported where it would appear that had the pulse been disregarded a fatal result would have ensued. Also in the fatal cases mentioned the pulse is described as having failed first in a large proportion of cases. There still remain also advocates of the two different methods of administration, the one upholding the "drop method" from the commencement (Dr. Overholt[17] describes a new bottle for this purpose), and the other advising getting the patient under the influence of the drug as quickly as possible by starting with large doses. All are agreed that there shall be a free supply of air during the administration.

[1] B. M. J., Feb. 17th, 1894. [2] Lancet, June 2nd, 1894. [3] Lancet, July 7th. [4] N. Y. Med. Jour., June 23rd. [5] Med. Record, April 14th. [6] Ther. Gaz., March 15th. [7] Lancet, May 26th. [8] Lancet, April 14th. [9] Ther. Gaz., Feb. 15th. [10] Med. Record, March 31st. [11] Ibid. [12] Med. Record, Feb. 24th. [13] Ibid April 21st. [14] Med. Week, July 13th. [15] Epit. B. M. J., Feb. 24th., [16] Hospital, April 28th, and March 12th. [17] Med. Record, Feb. 10th.

